# Effects of remote combine exercise-music training on physical and cognitive performance in patients with Alzheimer’s disease: a randomized controlled trial

**DOI:** 10.3389/fnagi.2023.1283927

**Published:** 2024-01-11

**Authors:** Ghazaleh Shokri, Fatemeh Mohammadian, Maryam Noroozian, Sadegh Amani-Shalamzari, Katsuhiko Suzuki

**Affiliations:** ^1^Department of Exercise Physiology, Faculty of Physical Education and Sports Science, Kharazmi University, Tehran, Iran; ^2^Department of Neurology, Roozbeh Hospital, Tehran University of Medical Science, Tehran, Iran; ^3^Faculty of Sport Sciences, Waseda University, Tokorozawa, Japan

**Keywords:** music-therapy, combined training, muscle endurance, dementia, mental readiness

## Abstract

**Introduction:**

This study aimed to investigate the effects of combined remote music and exercise training on the cognitive, psychological, and physical function of patients with Alzheimer’s disease (AD).

**Methods:**

Forty-one AD patients were randomly allocated to three groups, including control (C), training (T), and training with music (TM) groups. Participants were evaluated by cognitive and performance test batteries before and after the interventions. Both experimental groups performed 36 remote workouts in 3 months online via WhatsApp video call individually with the trainer. Training included simple and varied movements of all physical indicators. The number of sets began with two sets and progressively increased to one set every month, 5–10 repetitions per set. The overload was applied by reducing the break between sets every week. The TM group performed the same exercises while listening to Mozart and traditional Iranian songs.

**Results:**

We observed a significant main, group, time, and interaction effect on Romberg (*ηp*^2^:0.72), 30 s chair sit and stand (*ηp*^2^:0.75), and walking on steppe test (*ηp*^2^:0.63). Furthermore, there was a significant main time and interaction effect on push-ups (*ηp*^2^:0.43), sit and reach (*ηp*^2^:0.64), and MMSE (*ηp*^2^:0.76). In all variables, two experimental groups demonstrated substantial improvements than the C group (*p* < 0.01). In addition, the TM group (27.8%) showed a significant improvement compared to the C group (−6.4%) and the T group (12.2%) in MMSE.

**Conclusion:**

Combined remote training with listening to music as adjuvant treatment is an appropriate item to improve the cognitive and physical performance of Alzheimer’s patients, especially during the COVID-19 pandemic.

## Introduction

Alzheimer’s disease (AD) is the most common type of dementia; the primary symptoms include impairments in cognitive function such as recent memory, attention, language, visuospatial, and praxis. The patients also have difficulties in physical function and sleep quality. Although medications have limited these effects, they also have side effects, including nausea, anorexia, diarrhea, vomiting, and weight loss (DeLaGarza, 2003). Hence, non-pharmacological treatments such as exercise, social activity, mental challenges, and a healthy diet as prevention strategies with fewer side effects are recommended (Mendiola-Precoma et al., 2016; Nemoto and Suzuki, 2018).

Research has shown that exercise training effectively reduces the complications of all types of dementia (Santana-Sosa et al., 2008). In this regard, the positive benefits of resistance training on physical performance and limiting AD progression due to increased strength and muscle mass among old adults have been reported (Tracy et al., 1999; Bamman et al., 2003). On the other hand, aerobic training significantly improves activities of daily living, cardiorespiratory fitness, and a slower decline in cognitive function in patients with AD (Venturelli et al., 2011; Sobol et al., 2016). In general, a combination of strength and endurance training, combined training, is recommended for Alzheimer’s patients to get the maximum benefits (Parvin et al., 2020). However, due to the low effectiveness of physical activity on cognitive function, adding a mental task to exercise training, known as dual-task training, has more significant effects on the mind and physical fitness of Alzheimer’s patients (Dennis et al., 2009; Parvin et al., 2020). Researchers have shown that dual-task training has more positive effects, such as improving attention and accuracy, on Alzheimer’s patients rather than the exercise alone (Åhman et al., 2019; Nam and Kim, 2021).

Music therapy is an important, low-cost, and effective way to maintain and improve cognitive function and social behavior in Alzheimer’s patients (Raglio et al., 2014). Music therapy has no side effects, so patients and their caregivers can easily use it. Many studies have shown that music therapy can improve various cognitive and psychological aspects such as attention, memory, orientation, depression, and anxiety (Bruer et al., 2007; Ozdemir and Akdemir, 2009; Särkämö et al., 2014; Satoh et al., 2015). Individuals can exercise while music is playing or perform each one separately. A few research studies have been done on the combined effects of music therapy and exercise so far; in these studies, cognitive and exercise stimuli were not applied simultaneously. Recently, Higuti et al. (2021) reported listening to music and practicing physical exercise for 12 weeks; one session per week (25–30 min) did not significantly change functional or cognitive performance in institutionalized older adults with dementia (Higuti et al., 2021). In contrast, some studies that have used more frequency (twice a week) and duration (one hour) of exercise and music sessions reported improvements in cognitive, psychological, and motor abilities in patients with mild to moderate dementia (Satoh et al., 2014, 2017; Prinz et al., 2021). Therefore, a combination of music therapy and exercise as dual-task training can be an excellent option to improve AD patients’ physical and cognitive fitness.

Listening to music in healthy human can modulate many physiological responses even during exercise (i.e., heart rate, catecholamines, muscle activation), which leads to improved performance (Ballmann, 2021). Furthermore, listening to music during exercise has positive impact on psychological (i.e., mood, motivation) and psychophysiological (i.e., rate of perceived exertion, arousal) status, which may have ergogenic potential. Moreover, research showed that music increases activity in some portions of the brain such as left inferior frontal gyrus and insular cortex activation that are important for physiological arousal, emotion, and perception (Bigliassi et al., 2018). So, listening to music during exercise could activate these brain regions and lead to an increase in cognitive processing speed and organization of movement (Bacon et al., 2012). Therefore, it seems that simultaneous exercise and music through neural activation and arousal have augmentation physiological and psychological effects to improve cognitive and physical performance.

Given that the physical effects of exercise training and the mental function of music therapy are more pronounced, we hypothesized that the combination of these two stimuli has a synergic impact on improving AD patients’ physical and mental function. Due to the pandemic of COVID-19 infection, we conducted the study online with a video call for the safety of patients and their caregivers. Therefore, the present study aimed to investigate the effects of adding music to exercise training on the mental and physical fitness of Alzheimer’s patients.

## Materials and methods

### Study design

**Figure 1:**
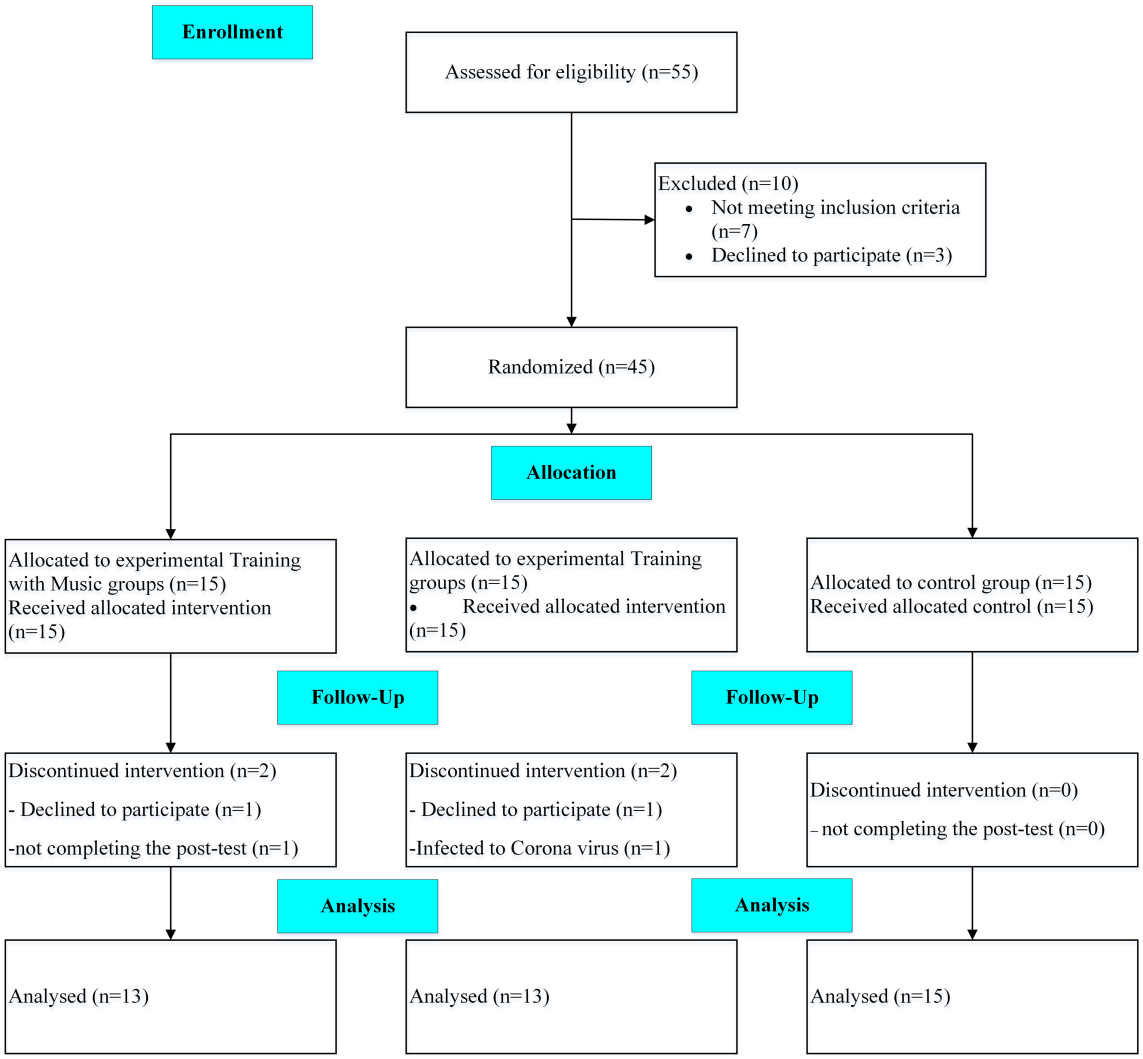
Schematic overview of study timeline (CONSORT flow diagram).

A randomized clinical trial, with control and two parallel experimental groups and a single-blind design, was conducted on AD patients from May 2021 until August 2021. This study aimed to investigate the effects of 12 weeks of adding music therapy to exercise training remotely on the physical and cognitive performance of patients with AD. The participants and their caregivers attended a familiarization session 1 week before the study. They were informed about the benefits and potential risks of the study, signed a consent form and cognitive and performance tests were taken. The block randomization method (size 6) was applied, and the participants were assigned to three groups. Because of the COVID-19 pandemic, the training sessions were executed online. A neurologist performed the cognitive test, and the trainer took the performance tests. A CONSORT flow diagram of the present study is shown in Figure 1.

### Participants

Patients with AD, who were eligible to participate in this Clinical Trials study, were recruited from the memory clinic of Roozbeh Hospital in Tehran, Iran. The inclusion criteria were AD patients aged 50–75 years with mild to moderate dementia and the ability to walk and perform exercise independently. A neurologist affirmed the diagnosis of dementia based on the Diagnostic and Statistical Manual of Mental Disorders (DSM-5) criteria, low to moderate stages of dementia based on the FAST scale (3–6), and laboratory tests. The patients’ medications were checked before beginning the study and were not changed in type or dosage during the intervention. In addition, patients with cardiac diseases (e.g., unstable angina and recent myocardial infarct) were not recruited. The exclusion criteria were the absence in more than three consecutive sessions, the unwillingness to continue training, the inability to perform exercises, not take post-tests, and the physician’s decision to exclude the patients. A sample size calculation was conducted using G*Power Software version 3.1.9.6 (Faul et al., 2007) for repeated measure ANOVA, using a rejection criterion of 0.05 and 0.8 (1-beta) power, and large effect (*f* = 0.5), a minimum of 13 participants needed to each group.

Forty-five eligible patients volunteered to attend the study, but the data of 41 patients (age: 67.5 ± 8.2 years; height: 169.4 ± 6.8 cm, body mass: 75.8 ± 7.7 kg) was analyzed finally. A third person who was not in the research team, based on the cognitive test scores, randomly assigned participants into three groups (*n* = 15), including training (T), training with music (TM), and the control group (C). Two participants from each experimental group were either not interested in continuing the intervention, infected with the coronavirus, or did not complete the post-test.

### Training protocol

The intervention lasted 3 months, 3 days per week (36 workouts). Each session was around 35–45 min, including 10 min of warm-up with dynamic movements, 20–35 min of main exercises, and 5 minutes of cool down with stretching movements. Because of the Coronavirus pandemic, all workouts were conducted online via WhatsApp video call individually with the trainer (researcher). The presence of a caregiver in the training session was mandatory. The training group performed a combination of sitting and standing movements. The movements involved the whole body and large muscle groups. The main exercises are presented in Table 1. In each session, exercises were selected from all indicators of physical fitness. The break between sets started at 70 s, and gradually it reduced 10 s every two weeks until the tenth week eventually reduced by 5 s to reach 25 s. The number of sets began with two sets and progressively increased to one set every month, 5–10 repetitions per set depending on the difficulty of the movement and the patient’s fitness. The trainer explained and demonstrated the activities to patients clearly. The TM group performed the same exercises while listening to the mix of Mozart’s sonatas and traditional and instrumental Persian music (Johnson et al., 1998; Li et al., 2015). We used the examiner-choose music playlist based on expert cognitive neurologist opinion, including some sort of Mozart music Symphonia, and also traditional Iranian music by the patient’s selection. Since familiarity with the music playlist could have positive effects on the patient’s cognitive function and probably mood improvements, we preferred to utilize the mix of classical Mozart music with established impact on cognitive function and a more familiar music playlist with possible familiarity evidence on cognition function. The session started with Mozart’s sonatas for 10–15 min, continued with Persian music, and then during the cool down, Mozart’s sonatas were played. The patient’s caregivers played music because of the greater clarity of the sound. Music was played throughout the exercise session. The overload was applied by reducing the break between sets every week (every three sessions). The training intensity was monitored using the Borg scale, a 10-point scale, in which we prescribed exercises in intensities of 4 to 6 on this scale. The C group did not receive any physical or cognitive intervention and continued their routine life; they participated in the pre and post-tests.

**Table 1:** Type of performed exercises.

Physical indicator	Kind of exercises
Balance	Keep balance on one foot, Heel-to-Toe
Coordination	Harmonious opening and closing of legs and arms, raise the opposite arm and leg simultaneously
Stretching	Reaching the fingertips to the toes while sitting on a chair, bringing the fingertips to toes while standing with straight knees
Upper body muscle endurance	Types of shoulder movements, shoulder press, biceps curl
Lower body muscle endurance exercises	Wall squat, raising feet simultaneously while sitting on a chair
Aerobic	Walking on the steppe, quick step

### Measurements

All the following tests were taken before the implementation of the research protocol and 48 h after the last training session. Initially, anthropometric measurements were taken, followed by cognitive tests, and finally performance tests.

### Anthropometric indices

Height and body mass were measured using a standard stadiometer (Seca 213, Germany) and a calibrated digital scale (Seca 769, Germany).

### Cognitive tests

Cognitive functions were assessed by the Mini-Mental State Examination (MMSE). The MMSE is a short exam to evaluate the quality of consciousness and has high sensitivity and specificity in detecting dementia across different age groups. This questionnaire included six subsets: orientation, registration, attention, recall, language, and constructional praxis, with a total score of 30 points. A score below 23 indicates the possibility of the disorder (Hobson, 2015).

### Performance tests

Physical performance tests were conducted in a written order with a 5 min interval. Before starting the testing, the participants had a 10 min warm-up, which included walking in place, chair sit-ups, wall push-ups, and light stretching. The Romberg, modified push-up, chair stand, sit and reach, and step tests were taken, respectively, to assess the patient’s physical performance.

The Romberg test for assessing a standing balance or participant’s ability to stand unassisted was performed on a firm and compliant support surface. Participants stood with both feet together without shoes, held their arms next to the body, or crossed in front of the body. Then, they were asked to close their eyes and keep their static balance. The time that the participants could stand with their eyes closed was recorded. The test was finished if participants opened their eyes, lost their balance by moving arms or feet, swayed their body, or began to fall and needed the examiner’s intervention to maintain balance (Forbes and Cronovich, 2021). Participants performed this test twice, and the best time was recorded.

The modified push-up is a test for the assessment of the patient’s shoulder endurance. In this test, the patients were asked to get push-ups positioned on their knees and do the push-up on their knees for 1 minute. The number of complete movements during 1 minute was counted.

We used the 30 s chair stand test to measure the subject’s lower body muscle endurance. The test needs participants to sit on a chair, put their hand on the opposite shoulder, and cross at the wrists; then, from the sitting position, subjects stand completely up and back down as many times as possible within 30 s. Count the total number of complete chair stands. This test was taken twice, and the best result was recorded for each patient (Rikli and Jones, 2013).

The sit and reach test measured the lower back and hamstring’s flexibility. The participants were asked to sit on the floor with their legs stretched out. The soles are placed flat against the box. Knees must be locked and pressed flat to the floor. With the palms facing downwards and the hands-on top of each other, participants reached forward and tried to touch or pass their toes. The participants reach out and hold that position for at least one-two seconds while the distance is recorded. The level of the feet was considered as recording zero so that any measure that does not reach the toes is negative and any reach past the toes is positive.

A step test measured the patient’s aerobic fitness. The participants stepped on and off the box (15–17 cm high) for two minutes, which was recorded by a chronometer. If patients start with the right or left foot, they should go down with the same leg with the “up,” “up,” “down,” and “down” rhythm. The number of complete steps on and off the box was counted for statistical analysis.

### Ethical aspect

All research procedures conducted in studies involving human participants were in accordance with the declaration of Helsinki. The study was approved by the Ethics Committee for Sport Sciences Research Institute of Iran (approval number: IR.SSRI.REC.1400.070). In addition, this research was registered in the Iranian Registry of Clinical Trials (IRCT) with registration number: IRCT20210726051993N1.

### Statistical analysis

The Statistical Package of Social Sciences (SPSS, IBM, v19) was used to analyze row data. Data presented in mean ± standard deviation (SD). The normality distribution of the variables was done using the Shapiro–Wilk test. A repeated measure analysis of variance ANOVA with the time (T1 vs. T2) and group (C, MT, T) was performed to analyze the data. We calculated the effect size (ES) by the change score divided by the SD of the change score to examine the magnitude of differences while controlling for the influence of the sample size (Dankel and Loenneke, 2018), with 0.2 considered as a small ES, 0.5 as a moderate ES and >0.8 as a large ES. The significance level was considered at *p* ≤ 0.05 for all statistical analyses.

## Results

### Cognitive performance

**Figure 2:**
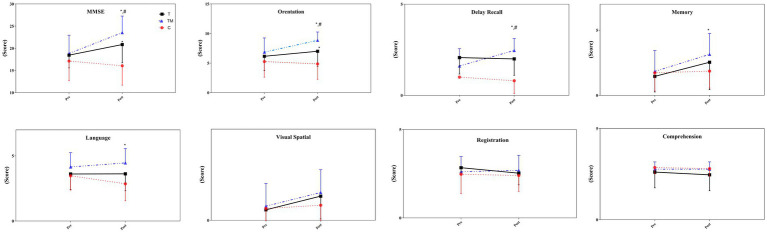
The scores of the MMSE test following the intervention. C, control group; T, training group; TM, training with the music group. *: significant difference with the C group; #: significant difference with the T group.

There was no significant difference between groups at the MMSE test at baseline (*p* > 0.05). The statistical analysis demonstrated there was no significant main group effect for MMSE (*F*_2,24_ = 3.50 *p* = 0.054, *ηp*^2^:0.23), through a significant time (*F*_1,12_ = 74.13 *p* = 0.001, *ηp*^2^:0.86) and interaction effect (group × time) (*F*_2,24_ = 38.71 *p* = 0.001, *ηp*^2^:0.76) observed. The TM group (27.8%) demonstrated a significant improvement compared to the C group (−6.4%) (*p* = 0.001), and the T group (12.2%) (*p* = 0.002). In addition, there was a significant difference between the T group and the C group (*p* = 0.001). In details, we observed significant differences at orientation (*F*_2,24_ = 9.74 *p* = 0.002, *ηp*^2^: 0.45), language (*F*_2,24_ = 3.52 *p* = 0.047, *ηp*^2^: 0.23), memory (*F*_2,24_ = 3.76 *p* = 0.042, *ηp*^2^:0.24) and delay recall (*F*_2,24_ = 5.59 *p* = 0.017, *ηp*^2^:0.32) between groups. However, there were not any significant differences at registration (*F*_2,24_ = 1.45 *p* = 0.257, *ηp*^2^: 0.11), visual-spatial (*F*_2,24_ = 1.32 *p* = 0.287, *ηp*^2^:0.10), and comprehension (*F*_2,24_ = 0.79 *p* = 0.412, *ηp*^2^: 0.06) following interventions between groups. For orientation and delay recall, there was a significant difference between the TM group and the other two groups (*p* < 0.05). Additionally, the scores obtained in the T group were significantly higher than in the C group in orientation (*p* = 0.013). The scores obtained in the TM group for language (*p* = 0.014) and memory (*p* = 0.035) were significantly higher than in the C group. Figure 2 displays the scores obtained from the MMSE test pre- and post-intervention.

### Physical performance

There were no significant differences between groups at physical performance tests at baseline (*p* > 0.05). Table 2 presents the descriptive statistics of body mass and performance parameters, pre- and post-intervention. There was a significant main time effect (*F*_1,12_ = 0.17.55, *p* = 0.001, *ηp*^2^:0.59), but no main group (*F*_2.24_ = 0.25 *p* = 0.779, *ηp*^2^:0.02), and interaction effect (*F*_2.24_ = 3.33 *p* = 0.053, *ηp*^2^:0.22) for body mass following intervention.

**Table 2:** The value of body mass and performance tests pre and after the intervention.

Variable	Group	Pre	Post	% change	Cohen’s *d*	*p* between group
Body mass (kg)	C	75.94 ± 9.09	74.91 ± 8.81	−1.33	−1.23	Pre: 0.711 Post:0.062
	T	76.23 ± 5.24	75.96 ± 5.49	−0.37	−0.27	
	TM	74.09 ± 6.27	73.74 ± 6.06	−0.45	−0.41	
30 s stand-up (N)	C	7.47 ± 2.32	6.40 ± 2.29	−11.15	−0.82	Pre: 0.512 Post:0.001
	T	8.53 ± 3.36	13.31 ± 4.29	64.46*	2.09	
	TM	8.62 ± 3.12	14.23 ± 3.92	75.07*	2.13	
Push-ups (N)	C	3.80 ± 3.80	3.20 ± 3.55	−16.22	−0.91	Pre:0.269 Post: 0.001
	T	5.54 ± 5.23	12.00 ± 10.33	183.52*	1.03	
	TM	6.92 ± 5.96	12.54 ± 9.87	100.09*	1.25	
Romberg (s)	C	11.40 ± 5.78	10.00 ± 5.39	−11.41	−1.10	Pre: 0.265 Post:0.001
	T	15.54 ± 8.38	31.31 ± 11.10	54.40*	1.97	
	TM	13.92 ± 5.63	34.08 ± 12.01	90.32*	2.07	
Step walking for 2 min (N)	C	16.73 ± 9.91	14.73 ± 9.61	−14.36	−1.62	Pre:0.089 Post:0.001
	T	26.00 ± 13.83	38.31 ± 20.44	59.52*	1.22	
	TM	25.54 ± 12.31	39.46 ± 13.78	74.27*	1.93	
Sit and reach test (cm)	C	−3.40 ± 3.85	−3.67 ± 4.56	3.05	−0.19	Pre:0.442 Post: 0.001
	T	−5.15 ± 3.80	−1.31 ± 4.66	−69.33*	2.01	
	TM	−4.31 ± 3.01	−1.07 ± 4.29	−44.17*	1.29	

Overall, compared to the C group, both experimental groups demonstrated substantial improvements in all performance indices following 12 weeks of training protocols. We found no significant main group effect for push-ups (*F*_2,24_ = 2.66 *p* = 0.091, *ηp*^2^:0.18), through a significant time (*F*_1,12_ = 32.56 *p* = 0.001, *ηp*^2^:0.73) and interaction effect observed (*F*_2.24_ = 9.20 *p* = 0.001, *ηp*^2^:0.43). The Bonferroni *post hoc* test showed the difference between the C group and both trained groups (*p* < 0.01). We observed a significant main group (*F*_2,24_ = 9.99 *p* = 0.001, *ηp*^2^:0.45), time (*F*_1,12_ = 103.55 *p* = 0.001, *ηp*^2^:0.90), and interaction effect (*F*_2.24_ = 30.41 *p* = 0.001, *ηp*^2^:0.72) for Romberg test. Two experimental groups demonstrated significant improvement than the C group (*p* < 0.01). For 30 s chair sit and stand, there was a significant main group (*F*_2,24_ = 6.18 *p* = 0.007, *ηp*^2^:0.34), time (*F*_1,12_ = 79.86 *p* = 0.001, *ηp*^2^:0.87), and interaction effect (*F*_2.24_ = 36.48 *p* = 0.001, *ηp*^2^:0.75). The difference was between the trained groups with the C group (*p* < 0.01). We found a significant main group (*F*_2,24_ = 4.2 *p* = 0.027, *ηp*^2^:0.26), time (*F*_1,12_ = 46.46 *p* = 0.001, *ηp*^2^:0.79), and interaction effect (*F*_2.24_ = 20.35 *p* = 0.001, *ηp*^2^:0.63) for walking on steppe test. The Bonferroni *post hoc* test displayed two trained groups performed far better than the C group (*p* < 0.01). There was no significant main group effect for sit and reach test (*F*_2,24_ = 0.06 *p* = 0.094, *ηp*^2^:0.01), through a significant time (*F*_1,12_ = 33.24 *p* = 0.001, *ηp*^2^:0.74) and interaction effect observed (*F*_2.24_ = 21.06 *p* = 0.001, *ηp*^2^:0.64). The Bonferroni *post hoc* test showed both experimental groups demonstrated further stretch than the C group (*p* < 0.001).

## Discussion

The primary aim of this study was to examine the effectiveness of adding music as a cognitive activity to exercise at home on the cognitive and physical performance of Alzheimer’s patients. The present study’s findings indicated both trained groups demonstrated considerable improvements in all performance indicators and cognitive function than in the C group. However, the TM group showed much more significant improvement in cognitive function compared to the T group alone. The TM group demonstrated substantial improvements in orientation, delay recall, memory, and language. Therefore, the research findings show that adding cognitive stimuli such as music during exercise training has synergistic effects potentially through neural activation and arousal the on improving mental and physical performance to gain further benefits from physical training.

Although both the T (12.2%) and TM (27.8%) groups showed improvements in cognitive function, measured by the MMSE test, a significant improvement was observed in the TM group. It seems that performing exercises simultaneously with music has caused a further improvement in cognitive function by activating brain cells in remembering the music and remote memories. Given that there is an overlap between the areas of musical memory and the areas spared in AD, it was shown that listening to music can preserve these areas. In this regard, minimal cortical atrophy and minimally impaired glucose metabolism were observed despite a non-significant change in Aβ deposition (Jacobsen et al., 2015). In addition, music activates a broad network in the brain: bilateral temporal lobes, superior temporal regions, parahippocampal gyri, caudal anterior cingulate cortex, and ventral pre-supplementary motor areas (Satoh et al., 2006; Jacobsen et al., 2015). Moreover, the positive effect of music on cognition could be interpreted based on impacts on arousal and evoking autobiographical memory, which was demonstrated in previous studies. That could improve self-consciousness, global cognitive function, and especially behavioral symptoms of dementia. In detail, the TM group showed significant improvement in orientation, delay recall, memory, and language compared to the C group. Therefore, simultaneously performing these two effective interventions by further perfusion and activating some areas of the mentioned brain could be effective in remembering events and maintaining cognitive performance. In contrast, Higuti et al. did not find any improvement in the cognitive function of patients after 12 weeks of exercise with music (Higuti et al., 2021). The methodological differences between our research and the former study may explain this contradiction. Our research was a dual-task in which music and exercise were performed simultaneously, but in Higuti’s research, they were conducted separately. Overall, performing exercise and listening to music simultaneously by activating the brain and maintaining muscle fitness effectively improves cognitive function and quality of life in Alzheimer’s patients.

Loss of muscle fitness due to muscle atrophy is a complication of AD that can lead to the inability to perform daily tasks, a poor psychological state, and ultimately a decline in quality of life (Burns et al., 2010; Lane et al., 2018). Our findings indicated that upper and lower body muscle endurance improved in both TM and T groups. Our training protocol involved many muscle endurance exercises for the upper and lower body, which were performed in several sets in all workouts; thus, expecting an improvement in muscle fitness was especially obvious in these patients whose physical fitness had considerably declined. Our finding has been supported by previous studies showing that a period of combined training, including resistance and aerobic exercises, significantly improved muscle strength and endurance in patients with cognitive impairment (Santana-Sosa et al., 2008; Langoni et al., 2019; Parvin et al., 2020). As there is a strong relationship between muscle atrophy and declined cognitive function (Burns et al., 2010), it was recommended that muscle maintenance exercises be included in AD patients’ training programs. Therefore, combined training with enhancing the strength and muscular endurance of AD patients can promote patients’ independence in performing routine activities and, to some extent, be effective in improving patients’ mood and quality of life.

AD is also associated with reduced cardiovascular fitness (Lane et al., 2018) due to a sedentary lifestyle. Our findings show that both TM and T groups demonstrated an improvement in aerobic fitness, which was measured by walking on the steppe. Activities such as walking steps, quick going back and forth, and repetition of movements improve aerobic fitness. In this regard, Parvin et al. (2020) showed that a 12 week combined training period significantly improves aerobic fitness (Parvin et al., 2020). Interestingly, increased cardiorespiratory fitness is associated with reduced brain atrophy in AD patients (Burns et al., 2008). Thus, aerobic exercise is as essential as resistance training for Alzheimer’s patients. The TM group showed a slightly better aerobic performance than the T group. Studies reported that the synchronization of music and exercise can result in improved running economy, efficiency, oxygen consumption and performance (Bacon et al., 2012; Terry et al., 2012). Therefore, exercising while listening to music, cardiovascular fitness can be improved through running economy.

One of the problems that Alzheimer’s patients encounter is an imbalance resulting from loss of muscle strength and destruction of nerve cells. Both experimental groups enhanced their balance following the research protocol. Participants performed many balance exercises during the intervention; thus, continuous activation of the proprioceptive sense caused the patients’ balance to develop. A previous study also confirmed that combined training had positive effects on the balance of Alzheimer’s patients and reduced the risk of falls (Santana-Sosa et al., 2008). The most non-significant difference was seen in balance between two training groups. It’s clear that cognitive processes, particularly attention are crucial when performing a balancing movement (Woollacott and Shumway-Cook, 2002). Hence, listening to music during exercise can activate the brain regions, involved in cognitive processing and organization of movement (Bacon et al., 2012); thus, this intervention can lead to the improvement of balance movements. In this regards, it has shown, listening to music improved postural balance in visually impaired adolescents (Maatoug et al., 2023).

Changes in body mass and composition need long-term manipulation of intake and expended calories. Although the current intervention lasted 3 months and is a good opportunity for changes in body weight, no significant change was observed following the interventions in all groups. In the present study, the amount of calories consumed was not calculated; however, it is evident that the calories expended in the exercise groups increased. Research has shown that Alzheimer’s patients progressively lost their appetite due to hypo-perfusion to the right and left anterior cingulate and orbitofrontal cortices, ultimately leading to weight loss (Ismail et al., 2008). In the C group, we observed slightly more weight loss than in the two experimental groups, which indicates that exercise training has effectively restored patients’ appetite.

We acknowledged that there were some limitations in our study. Firstly, we had limitations in using laboratory tools due to the coronavirus pandemic; we did not measure patients’ body composition, which can be used to interpret the findings. Secondly, we also did not assess the changes in brain imaging after intervention due to the pandemic crisis. Hence, we propose evaluating the correlation between cognitive changes and alteration in brain structure by utilizing brain MRI and functional brain imaging such as brain Single Photon Emission Computed Tomography (SPECT) and Positron emission tomography (PET) scan and measuring body composition by dual energy X-ray (DEXA) in future studies. Additionally, we suggest that future studies evaluate the effects of the dual-task intervention on behavioral and psychological symptoms of dementia, sleep problems, and eventually the quality of life in dementia patients. In addition, given that familiar and unfamiliar music may have different effects on cognitive and performance indicators, it is recommended that researchers investigate this issue in a research. At last, in follow-up studies after 3–6 months could help us determine the constant effect of dual-task training on cognition.

## Conclusion

In conclusion, our findings showed a combining remote training with music as appropriate non-pharmacological treatment is useful for improving the physical and cognitive function of Alzheimer’s patients and could ultimately decrease the AD burden on society. Thus, we recommend that neurologists include dual-task training (training with music) in the adjunctive treatment of Alzheimer’s patients, along with pharmacological therapies.

## Data availability statement

The datasets presented in this article are not readily available because the datasets generated and analysed during the current study are available from the corresponding author on reasonable request. Requests to access the datasets should be directed to amani_sadegh@khu.ac.ir.

## Ethics statement

The studies involving humans were approved by Sport Sciences Research Institute of Iran. The studies were conducted in accordance with the local legislation and institutional requirements. The participants provided their written informed consent to participate in this study.

## Author contributions

GS: Data curation, Methodology, Writing – original draft. FM: Conceptualization, Supervision, Writing – review & editing. MN: Conceptualization, Writing – review & editing, Formal analysis. SA-S: Conceptualization, Formal analysis, Writing – review & editing, Supervision. KS: Writing – review & editing.
